# An End-to-End Deep Reinforcement Learning-Based Intelligent Agent Capable of Autonomous Exploration in Unknown Environments

**DOI:** 10.3390/s18103575

**Published:** 2018-10-22

**Authors:** Amir Ramezani Dooraki, Deok-Jin Lee

**Affiliations:** Smart Autonomous Systems Lab, School of Mechanical & Automotive Engineering, Kunsan National University, Gunsan, Jeonbuk 54150, Korea; a.ramezani.dooraki@gmail.com

**Keywords:** deep reinforcement learning, autonomous agent, adaptive agent, autonomous exploration, obstacle avoidance, bio-inspired

## Abstract

In recent years, machine learning (and as a result artificial intelligence) has experienced considerable progress. As a result, robots in different shapes and with different purposes have found their ways into our everyday life. These robots, which have been developed with the goal of human companionship, are here to help us in our everyday and routine life. These robots are different to the previous family of robots that were used in factories and static environments. These new robots are social robots that need to be able to adapt to our environment by themselves and to learn from their own experiences. In this paper, we contribute to the creation of robots with a high degree of autonomy, which is a must for social robots. We try to create an algorithm capable of autonomous exploration in and adaptation to unknown environments and implement it in a simulated robot. We go further than a simulation and implement our algorithm in a real robot, in which our sensor fusion method is able to overcome real-world noise and perform robust exploration.

## 1. Introduction

The ability to learn is an important aspect of intelligence. A robot with this ability is capable of learning from its surrounding environment using its own experiences, autonomously and continuously, similar to intelligent creatures that can be found in nature. This kind of robot can adapt itself to its environmental changes and maximizes its long-term intrinsic and extrinsic rewards or, in other words, answers its intrinsic and extrinsic motivations. With the aim of achieving such an ability and by considering the recent advances and progress in the area of artificial intelligence, the creation of robots that are able to autonomously train and adapt themselves to their surrounding environment is now possible. The result is having robots not only in industrial uses such as factories but also as entities in our everyday social life—an idea that is no longer a fantasy.

Some important capabilities that allow robots to achieve a high degree of autonomy are autonomous exploration and navigation. Looking at the literature, there are a couple of works that tried to accomplish these capabilities using methods such as Ant Colony Optimization (ACO) [1], Genetic Algorithm (GA) [2], Particle Swarm Optimization (PSO) [3], Fuzzy Neural Network (FNN) [4], Learning from Demonstration (LfD) [5] and Reinforcement Learning (RL) [6]. However, in this paper we are interested in methods that are based on machine learning methods and specifically RL [7]. For example, Lee et al. [8] trained a network for their RL agent so their quadruped robot could avoid obstacles. In Kominami et al. [9], an RL agent is used in combination with virtual repulsive method by a multi-legged robot to tackle obstacles using its tactile sensors. Zhang et al. [10] used model predictive control (MPC) to train a RL agent to learn to fly a quad-copter; MPC is necessary for training but not necessary for the testing phase. In Zhang et al. [5], the Gaussian mixture model (GMM) and Gaussian mixture regression (GMR) are used for learning from demonstration LfD with the goal of avoiding obstacles. Sadeghi and Levine [11] used deep reinforcement learning (DRL) to make a drone explore and avoid obstacles using a monocular camera. A Deep Q-Network (DQN) [12]-based method was used in [6,13] to train their robot to explore autonomously and avoid obstacles while they used initialized weights for their network (weights are generated using a supervised learning method). Smolyanskiy et al. [14] used off-the-shelf hardware to design a deep neural network (DNN) called TrailNet for following a trail. In [15], we have developed an algorithm that learns from scratch and in an autonomous way using RL and Multi-Layer Perceptron (MLP) to explore autonomously. Some of the aforementioned works are using mono-chrome cameras and use a network in front of the mono-chrome camera to convert the RGB image to a depth image. Some other works use external devices such as Vicon system for the generation of reward and state, which emphasizes the fact that the system needs the external systems for training or even working. Furthermore, there are works that use DNNs, and works that use continuous action space, and works that use a real robot in order to implement their algorithms. Nonetheless, works that use a combination of all the aforementioned advantages are harder to find. Thus, our purpose is to design and implement an algorithm that works with DNNs, without the help of external systems and by using a sensor fusion that provides noise-resistant data for our robot so our algorithm can focus on learning autonomously to explore and avoid obstacles using discrete and continuous actions.

In this paper, we developed an enriched new algorithm based on our previous work [15]. Here we used a memory-based DRL method in our development and, as a result, we call our new algorithm Memory-based Deep Reinforcement Learning (MDRL). Comparing our previous algorithm to MDRL, the main differences are in the input of our algorithm, in which there is a high dimensional space—a three-dimensional matrix of { 8 × 80 × 60 }—while the previous one is a vector of {80} elements’ and the capability of using both discrete and continuous actions in MDRL. In addition to simulating our robot to prove the capability of our algorithm, we also implement it in a real robot. As we explain in the following, our algorithm is capable of learning by itself from its own experiences in an end-to-end fashion to explore and avoid obstacles autonomously. We fuse our depth sensor with our range sensors as we explain later, but we do not pre-process the input sensor data or implement any kind of mathematical filters or convolution on it. Despite that, compared to many of the papers and algorithms in the literature, our algorithm is able to adapt itself to changes in the environment due to sensors that are used to generate the reward for our system. Furthermore, our algorithm continues updating its policy or, in other words, adapts itself even when an optimal policy is discovered. The other difference is that our work starts learning from scratch and we do not use initialized weights for our training.

Our work is inspired by the ideas mentioned in [12,16] and previously in [7] for the creation of our function approximator, which is used in our reinforcement learning-based algorithm. In the following we emphasize the achievements of our algorithm and work:

- Our algorithm uses the DRL method for learning from scratch to explore and avoid obstacles in an unknown environment, in an end-to-end fashion or, in other words, without preprocessing our sensor data, in an autonomous, continuous and adaptive way.
- While our proposed algorithm in this paper is able to use discrete actions for exploration and obstacle avoidance, we enhanced our previous work even more in order to be able to work in a continuous action space as well.
- Our algorithm benefits from having a memory-based implementation, it has a long-term and a short-term memory, which allows our robot to be able to distinguish between similar states and being able to learn from its own long-term experiences.
- We use a centralized sensor fusion technique in order to overcome the noise that exists in a real-world scenario and to be able to recognize the environment in a robust way.
- Our algorithm is tested in a simulated robot; nevertheless, in this paper, we moved further and implemented our algorithm in a real robot.

In the rest of this paper, we first explain about our main algorithm and the different aspects of its implementation considering our previous work [15], and we focus on the enhancements and necessary explanations. After that, we explain about our testing, simulation and real test. Finally we discuss our results and conclude our work.

## 2. Memory-Based Deep Reinforcement-Learning (MDRL)

**Figure 1:**
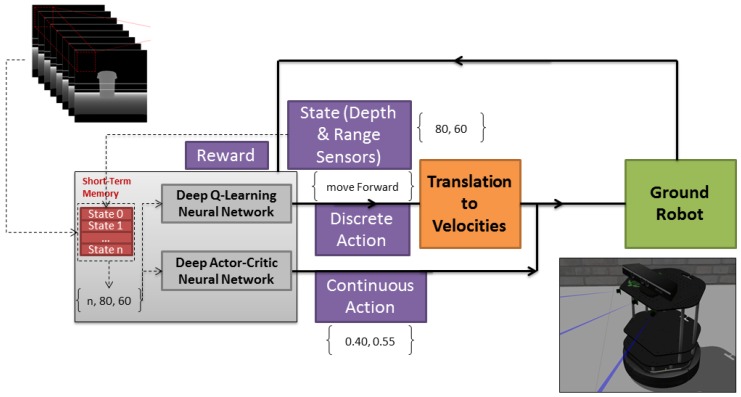
MDRL algorithm work flow.

Figure 1 shows the work flow of our algorithm (Algorithm 1). The input to the algorithm is our sensor data which encompasses a depth sensor and range sensors. We consider our problem of autonomous exploration as a Markov Decision Process (MDP), and we use the RL family of algorithms to solve it. Using RL to solve our problem means learning to take the best decision at time *t* in state $s_{t}\in S$. The best decision will be taken according to the policy that is learned by the algorithm. After executing an action, the state of the algorithm will be changed to a new state. An epsilon-greedy policy is used in our algorithm, which decides which action is the best according to the value of each action in a particular state where it is calculated by our DNN module. Using DNN instead of a q-table (in a conventional RL agent) will allow our RL to be able to generalize and approximate an optimal action or, in other words, an optimal policy $\pi^{*}$ considering the state of our agent. As a result, instead of searching our q-table for the action with the highest value, we use DNN to approximate for us what action has the highest possible value, using DNN also to allow our robot to generalize and take proper action on states it did not encounter before. Finally, considering the intrinsic and extrinsic motivation of our robot that is explained later in Reward section, our algorithm generates a reward *R* and updates our DNN when our robot is situated in state $s_{t+1}\in S$.

**Algorithm 1** Memory-based Deep Reinforcement Learning (MDRL) Algorithm1:**procedure**Sensor_Fusion(DepthImage,RangeSensors)2:    **if** (each RangeSensors Value <0.5 m) **then**3:        DepthImage[at the related positions] = Particular RangeSensors Value4:    **end if**5:    return DepthImage6:
**end procedure**
7:**procedure**Reward(RangeSensors,Action,Action_Type)8:    $R_{t_{external}}=\left(RangeSensor_{left}\right)+\left(RangeSensor_{center}\right)+\left(RangeSensor_{right}\right)$9:▹ Range Sensor values are scaled to be in range[0, 1.0], originally were [0, 200] cm10:    $A_{forward}=0.0,A_{turning}=0.0$11:    **if** (Action_Type==Discrete_Action) **then**12:        **if** (Action == forward) **then**: $A_{forward}=1$13:        **else if** (Action == turning) **then**: $A_{turning}=1$14:        **end if**15:    **else if** (Action_Type==Continuous_Action) **then**16:        $A_{forward}=Action\left[0\right]$▹ Action[0] = real value generated for linear_velocity_x of robot17:    **end if**18:    $R_{t_{intrinsic}}=A_{forward}+\left(-1.0\times A_{turning}\right)$19:    return $R_{t_{external}}+R_{t_{intrinsic}}$20:
**end procedure**
21:
**procedure**
Get_State()
22:    s[1…n]←s[0…(n−1)]23:    s[0]←SENSOR_FUSION()24:    return s25:
**end procedure**
26:**procedure**Main(DepthImage,RangeSensors)▹ main procedure27:    Initialize Action_Type28:    **if** (Action_Type==Discrete_Action) **then**29:        Randomly initialize deep network $Q_{deep}\left(s,a|\theta^{Q_{deep}}\right)$30:        Initialize deep target network $Q_{deep}^{\prime }$ with weights $\theta^{Q_{deep}^{\prime }}\leftarrow \theta^{Q_{deep}}$31:        **while**
i<infinity**do**▹ this procedure continues for ever32:              $s_{t}\leftarrow Get\_State\left(\right)$33:              Select Action $a_{t}$ according to ϵ−greedy Policy34:▹$a_{t}=a_{max}Q_{deep}\left(s_{t},a_{t}|\theta^{Q_{deep}}\right)$ or Random Action Considering an Annealing ϵ35:              Wait for robot to move to the new position36:              $s_{t+1}\leftarrow Get\_State\left(\right)$37:              $r_{t}\leftarrow Reward\left(\right)$: Store transition $\left(s_{t},a_{t},r_{t},s_{t+1}\right)$ in DeepReplayMemory38:              Set $y_{i}=r_{i}+\gamma Q_{deep}^{\prime }\left(s_{i+1},a|\theta^{Q_{deep}^{\prime }}\right)$39:              Update deep Q -network by minimizing the loss: $L=\frac{1}{N}\Sigma_{i}{\left(y_{i}-Q_{deep}\left(s_{i},a_{i}|\theta^{Q_{deep}}\right)\right)}^{2}$40:              Update the deep target network, $Q_{deep}^{\prime }\leftarrow \tau \theta^{Q_{deep}}+\left(1-\tau \right)\theta^{Q_{deep}^{\prime }}$41:        **end while**42:    **else if** (Action_Type==Continuous_Action) **then**43:        Randomly initialize deep Critic network $Q_{deep}\left(s,a|\theta^{Q_{deep}}\right)$ and deep Actor network $\mu_{deep}\left(s|\theta^{\mu_{deep}}\right)$44:        Initialize deep target network $Q_{deep}^{\prime }$ and $\mu_{deep}^{\prime }$ with weights $\theta^{Q_{deep}^{\prime }}\leftarrow \theta^{Q_{deep}}$, $\theta^{\mu_{deep}^{\prime }}\leftarrow \theta^{\mu_{deep}}$45:        **while**
i<infinity**do**▹ this procedure continues for ever46:              $s_{t}\leftarrow Get\_State\left(\right)$47:              Select action $a_{t}=\mu \left(s_{t}|\theta^{\mu }\right)+N$48:▹N=Random(Gaussian)Noisewithmean=0.0andstd=0.249:              Wait for robot to move to the new position50:              $s_{t+1}\leftarrow Get\_State\left(\right)$51:              $r_{t}\leftarrow Reward$: Store transition $\left(s_{t},a_{t},r_{t},s_{t+1}\right)$ in DeepReplayMemory52:              Set $y_{i}=r_{i}+\gamma Q_{deep}^{\prime }\left(s_{i+1},\mu^{\prime }\left(s_{t+1}|\theta^{\mu^{\prime }}\right)|\theta^{Q_{deep}^{\prime }}\right)$53:              Update deep Critic network by minimizing the loss: $L=\frac{1}{N}\Sigma_{i}{\left(y_{i}-Q_{deep}\left(s_{i},a_{i}|\theta^{Q_{deep}}\right)\right)}^{2}$54:              Update deep Actor network by using the sampled policy gradient:55:              $\nabla_{\theta \mu }\approx E_{s_{t}∼\rho^{\beta }}\left[\nabla_{a}Q\left(s,a|\theta^{Q}\right){|}_{s=s_{t},a=\mu \left(s_{t}\right)}\nabla_{\theta_{\mu }}\mu \left(s|\theta^{\mu }\right){|}_{s=s_{t}}\right]$56:              Update the deep target networks:57:              $\theta^{Q_{deep}^{\prime }}\leftarrow \tau \theta^{Q_{deep}}+\left(1-\tau \right)\theta^{Q_{deep}^{\prime }},\theta^{\mu_{deep}^{\prime }}\leftarrow \tau \theta^{\mu_{deep}}+\left(1-\tau \right)\theta^{\mu_{deep}^{\prime }}$58:        **end while**59:    **end if**60:
**end procedure**


### 2.1. Control and Decision-Making

In this algorithm we have used the Q-Learning [17] agent from the family of RL model-less agents for the purpose of the robot decision-making, in order to allow the robot to learn an optimal policy for autonomously controlling its movements due to the following facts:

- Our algorithm does not have a model of the problem (environment)
- Using long-term memory by implementing a method mentioned in [18], a model-free RL agent is needed

In order to find a solution and make our algorithm work successfully, we need to train our DNN in a way that finds the action that maximizes our reward in each particular state. As a result, the final outcome of our algorithm will be an optimal policy (a trained DNN) that is able to choose the best action $a_{t}\in A$ in state $s_{t}\in S$.

### 2.2. Discrete Action

Our algorithm creates a loop in which in each of its iterations, an epsilon-greedy policy will take an action and receives a reward *R* and goes to state $s_{\left(t+1\right)}\in S$. At this time, we use the future discounted reward using the Bellman equation with α=0 to update our policy. Since our optimal policy is in fact our DNN we need a loss function and also a method to reduce the loss. For this purpose we use a similar loss function to that mentioned in [12] and use Stochastic Gradient Descent (SGD) algorithm to reduce the loss of our DNN. Our q-function approximator is **$Q(s_{t},a_{t};\theta )$** and we try to reduce its difference with the following value, which we call *y*:

(1) $$\begin{array}{c} E_{s_{t+1}}[Q\left(s_{t},a_{t};\theta \right)+\alpha .(R+\gamma .max_{a}Q\left(s_{t+1},a;\theta_{target}\right)\\ -Q\left(s_{t},a_{t};\theta \right))|s_{t},a_{t}] \end{array}$$

### 2.3. Continuous Action

For continuous action space, we use a family of RL algorithms called Actor-Critic. We use an Actor model at time *t* to take action $a_{t}$ in state $s_{t}$, and this action is generated using a deterministic policy π:S↦P(A) and run in the environment *E*. Next, we observe the new state $s_{t+1}$ and use our Critic network in order to measure how good our action selected by our Actor was in state *s*. In order to implement our method, we used an algorithm called Deep Deterministic Policy Gradient (DDPG) and since we use a high-dimensional input as our state, we used the DDPG-Pixel-based algorithm. In DDPG we generate the action using $a_{t}=\mu \left(S_{t}\right)=\mu \left(st|\theta_{t}^{\mu }\right)+N$. *N* is the noise we generate purposely in order to let our algorithm do stochastic exploration, and this exploration will allow our algorithm to explore the action space, $A=R^{N}$. For generating our noise, we used random samples selected from a normal (Gaussian) distribution with mean=0.0 and std=0.2.

(2) $$L\left(\theta^{Q}\right)=E_{\left(s,a\right)}\left[{\left(Q\left(s_{t},a_{t};\theta^{Q}\right)-y_{t}\right)}^{2}\right]$$

where:

(3) $$y_{t}=R+\gamma .Q(s_{t+1},\mu \left(s_{t+1}|theta_{Q}\right)$$

The DDPG-Pixel algorithm uses a parameterized Actor model which specifies the current policy. Furthermore, it has a Critic model that is trained using the Bellman equation, similarly to Q-Learning. The Actor parameters will be updated using the chain rule and by the gradient of Critic model:

(4) $$\nabla_{\theta \mu }\approx E_{s_{t}∼\rho^{\beta }}\left[\nabla_{a}Q\left(s,a|\theta^{Q}\right){|}_{s=s_{t},a=\mu \left(s_{t}\right)}\nabla_{\theta_{\mu }}\mu \left(s|\theta^{\mu }\right){|}_{s=s_{t}}\right]$$

### 2.4. Sensor Fusion Technique

**Figure 2:**
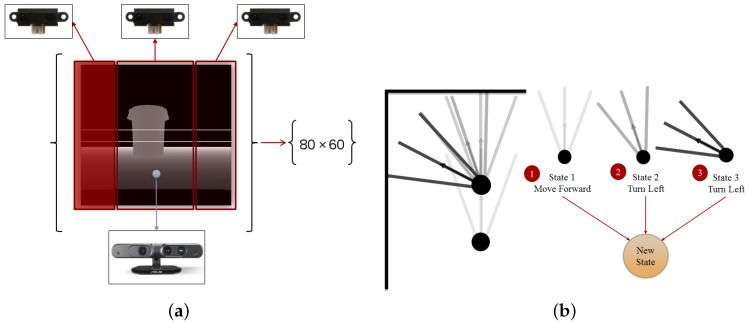
Sensor fusion and state generation. (**a**) Sensor fusion and state generation; (**b**); In order to enhance our robot capability, we combine several states and make a new state which in fact shapes our short-term memory (an example of memory with size three), adapted from [15].

With the idea of using our robot for a real purpose and in a real environment, we considered the noise effect on our depth sensor. In a simulated environment it is possible to reduce the noise to zero, but in a real-world scenario it is not possible. As a result, and in order to overcome the noisy depth image specifically when our robot is near to an object, a typical depth sensor is only able to measure a depth of 50 cm to 5 m, we fused our depth sensor with three range sensors and tested it in a simulated robot and later in a real robot. It is important to attach the range sensors in a way that cover all the horizontal view of the depth sensor, and furthermore it is necessary to attach the range sensor in the same position on the simulated and real robot. In this work, we installed the sensors at three different angles (directions); −30°, 0° and +30°. Each sensor covers a specific part of the depth sensor, and if an object comes to a distance less than 50 cm, we change the depth sensor output by using the range sensor values in corresponding areas of the depth image (Figure 2a).

### 2.5. Memory-Based Method

**Figure 3:**
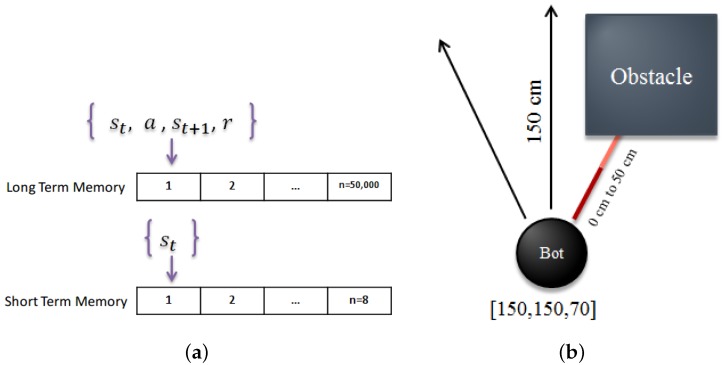
Memory and reward calculation. (**a**) Long-term and short-term memory used in our algorithm; (**b**) Range sensors used for reward calculation, adapted from [15].

In almost every kind of intelligent creature, a memory structure can be found which either acts like a simple buffer or like a complicated memory structure with short-term and long-term properties. One of the main reasons for having a memory is to remember what happened in the past in order to choose the best action in the future. For our robot, we used two types of memory which can be seen in Figure 3a. One type is a memory replay based on [18] mechanism, which contains all the last 50,000 moves of our robot. This memory replay can be interpreted as a long-term memory for our robot, where it can look at it and see the result of a behavior in a certain condition. Here we use this long-term memory in a similar way to the DQN in [12] by randomly selecting a batch of experiences from the memory for training our DNN. The second memory is a short-term memory which contains the last n movements of our robot. As can be seen in Figure 2b, we generate a super state, which encompasses n latest moves of the robot, and we interpret this super state as our short-term memory. Having a short-term memory helps our robot to distinguish the differences between similar states. As a result, our new state can be obtained from the following formula:

(5) $$S_{memory-based}=\left[S_{1},S_{2},\ldots ,S_{n}\right]$$

### 2.6. State Perception

Considering the methods mentioned in the past two sections, sensor fusion mechanism and memory-based state, our algorithm perceives the state of the robot by processing the fused data of depth and range sensors (Figure 2a). In this paper, in order to increase the speed of our training and reduce the necessary hardware capabilities, especially considering our real robot scenario, we resized the result of our fusion section in order to achieve a depth image of size 80 by 60 pixels. Furthermore, having used a memory-based method, we use n depth image to generate our state (Figure 2b). *n* can be any number but increasing n more than 16 can increase the training time drastically, thus we tested our algorithm with *n* = 1, 5 and 8 for discrete action-based method and *n* = 1 for continuous action-based.

### 2.7. Agent Reward

A RL agent is able to autonomously learn the best action in a specific state and this happens by receiving the proper reward after executing each action. Thus, when the agent is behaving correctly it should receive positive rewards. Receiving the correct rewards in the corresponding state helps the RL agent to estimate how good or bad its action is. Thus, it is crucial to define the correct and only necessary rewards. Considering Figure 3b, we use our range sensors to measure the robot distance with the nearest obstacles and generate the proper reward. When the agent is very near to an obstacle, it receives a negative reward. Furthermore, in any situation, if our agent rotates left or right it receive a small negative reward, and if it moves forward when there is no obstacle in front, it receives a small positive reward. These negative and positive rewards can be considered as an intrinsic motivation that gives the agent enough tendency to move forward when it is possible. The following equation shows the reward calculation; In terms of “Discrete Action” $A_{forward}$ and $A_{turning}$ are either 0.0 if not selected and 1.0 if selected, nonetheless, for Continuous Action $A_{turning}$ is always 0.0 and $A_{forward}$ is equal to the output of the first element of Actor network—Action[0] which is a real value in range[−1, 1].

(6) $$\begin{array}{c} R_{t_{external}}=\left(Sensor_{l}\right)+\left(Sensor_{c}\right)+\left(Sensor_{r}\right)\\ R_{t_{intrinsic}}=A_{forward}+\left(-1.0\times A_{turning}\right)\\ R=R_{t_{external}}+R_{t_{intrinsic}} \end{array}$$

### 2.8. Deep Neural Network

**Figure 4:**
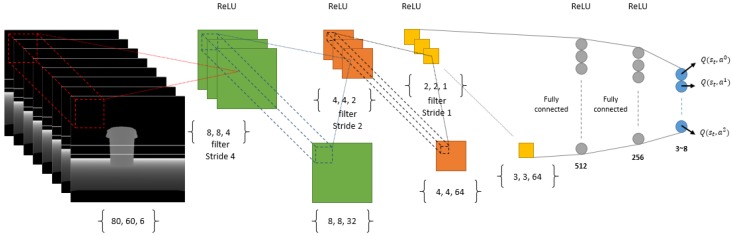
The deep learning architecture used as a function approximator in our deep reinforcement learning regarding discrete-action type by changing the DQN ([12]) architecture slightly to suit our input and short-term memory structure.

**Figure 5:**
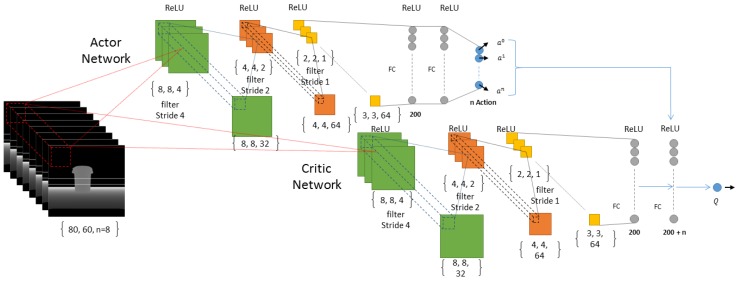
The deep Actor-Critic architecture used as a function approximator in our deep reinforcement learning regarding Continuous-Action type by changing the DDPG ([16]) architecture slightly to suit our input and short-term memory structure.

We have used a DNN as the function approximator with in our RL module. The reason we are using a function approximator is mainly because of the number of states we can have. Imagine our state is a matrix of 80 by 60 dimensions where each dimension can have a scalar value from 0 to 255, so the total number of states for a memory of size 1 (that is only one image as our state) will be about ${\left(80\times 60\right)}^{256}$ which is a high-state dimensionality. As a result, we need to use a function approximator such as a DNN in order to be able to manage the states by doing generalization and enhance it over time using more samples than it receives from its own experiences. For our discrete-action scenario we use a convolutional neural network with {80×60×n} inputs where *n* is the number of images in short-term memory. In our experiments we used 1, 5 and 8 for *n* and finally decided to use n=8 (Figure 4) because it shows a better result in comparison to 0 and 5, as can be seen in the results of our simulation section. The input to our deep architecture is actually our super state (with a memory of size 8) and 3 outputs corresponding to the value of all three possible actions (the number of actions can be increased according to the necessities, but more actions need more time for training and as the number of actions increases, convergence of the policy to optimal will be harder, mathematically) in each state which are rotate left, rotate right and move forward. Furthermore, it has three convolutional layers and two fully connected hidden layers with 512 and 256 neurons. Our neurons activation functions are Relu in all layers, except the last one, which is Linear (Figure 4). We defined Adam optimization method for our DNN with a learning rate of 0.001. For our continuous-action case, we used a deep architecture similar to the discrete-action case in terms of network architecture. Nonetheless, the fully connected layers are two layers, each one with 200 neurons which shape our Actor network and, in Critic, the output of our network is the only Q value and the output of Actor network is concatenated with the second fully connected layer of the network. Our actions consist of a turning range of [−1, 1] and gas (moving forward) range of [0, 1] which is the output of our Actor network as can be seen in Figure 5. Finally we used memory-reply mechanism [18] for optimal training of our DNNs.

## 3. Simulation

### 3.1. Environment

**Figure 6:**
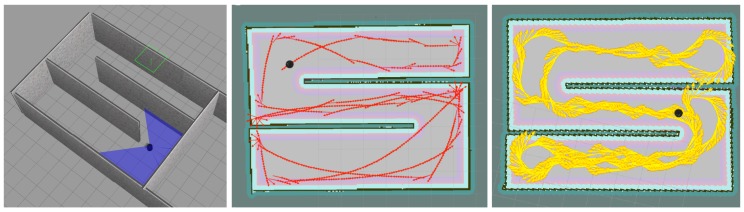
From (**left**) to (**right**), first image shows the robot simulated in Gazebo in a simple environment. The second image shows the robot movements using discrete actions visualized by SLAM after robot learned an optimal deep Q-Learning policy autonomously. The third image shown the robot movements using continuous actions visualized by SLAM after robot learned an optimal deep Actor-Critic policy.

For our simulation we have used Gazebo simulator [19] and a robot model called Turtlebot as our base model. Since we had the idea of testing our robot in a real scenario we changed the provided Turtlebot model in Gazebo sim to match an actual robot provided from [20]. Firstly, We changed the depth camera position, and secondly, we added three range sensors to our robot model in Gazebo sim (Figure 1 (simulated robot image)), these range sensors are important considering our explanation in ‘State Perception’ and ‘Agent Reward’ sections. Furthermore, we used a Robot Operating System (ROS) [21] for the purpose of connectivity between our simulated robot and our algorithm, written in Python. Using ROS provides us with defined nodes and topics for each module, which we use for controlling the robot and also receiving information from it using the ROS connectivity protocol. Using ROS can give us the re-usability benefit of using the same code with minor changes for a real robot. Figure 6, left, shows the robot simulated in a simple training environment in Gazebo.

### 3.2. Result

In order to fully check the capability of our algorithm, we performed several tests using our simulated robot (Figure 1) in Gazebo where we defined our short-term memory sizes to be 1, 5 and 8 and our long-term memory of size 50,000 for discrete-action space and with no short-term memory (since the robot is capable of choosing the degree of rotating, it is able to pass the temporal limitation that exists in our discrete-action space) and long-term memory of 20,000 for our continuous-action space. Furthermore, we first used our algorithm to train our robot in a simple environment (Figure 6 (left)), and later we will move our trained robot to a complex environment in order to test its adaptability.

**Figure 7:**
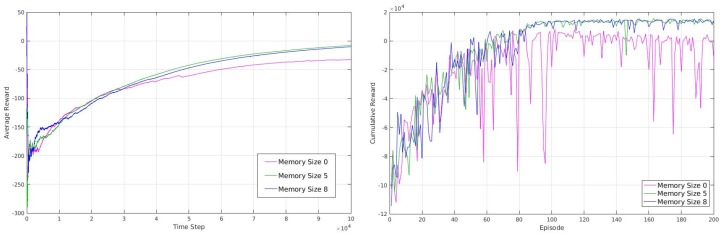
The (**left**) graph shows the average reward that robot obtains using Deep Q-Learning (discrete-action) over time (showing that robot is able to maximize its average reward). The (**right**) graph shows the cumulative reward that robot accumulate over time and in each episode, the graphs are drawn over (100,000 steps) using no short-term memory, short term memory of size 5 and short-term memory of size 8.

In a normal RL case, a typical agent learns in an episodic way, where after each episode (training cycle), the robot position will be reset to a random position. In our case, however, we did not use an episodic scenario, and as a result our robot learned continuously without being reset to a random position. The benefit of this method is that it allows the robot to learn to release itself from corners (which can be considered as termination states in episodic approach). In fact, in a normal social environment, the robot does not have the possibility of resetting its position so it needs to learn to quit from a corner or other challenging situations. Figure 7 (left) is the average reward gathered by our robot in discrete-action space over 100,000 steps, using different sizes of short-term memory. As we mentioned earlier, we do not train our robot in an episodic way; however, we calculate the cumulative reward gathered by our robot every 500 steps in order to achieve a second measurement of our robot behavior shown in Figure 7 (right).

Considering the cumulative rewards calculated over each of the 500 steps, we can see there is a big difference between the robot behaviors while having no short-term memory and having a short-term memory of size 5 or 8, where the robot reward maximization is stable with a short-term memory of size 5 and 8 but is not stable with no short-term memory. This unstable behavior of the robot (inability to maximize the reward in a stable way) arises from the fact that the robot with no memory sticks to some moments in corner positions and does repetitive behavior that results in gathering negative rewards. In term of average reward, as can be seen, it is higher for short-term memory of size 5 and 8 compared to short-term memory of size 0. In our experiments, we used an epsilon of 1.0 for the beginning that annealed to 0.1 by reaching to 50,000 steps and with no change from 50,000 to 100,000. As a result, the average reward obtained by robots with different sizes of short-term memory is more similar up to 50,000 steps because of random movements and starts to show a clearer difference after about 50,000 steps where epsilon is 0.1. As can be seen, increasing the size of short-term memory to more than 5 contributes very little to the increment or robustness of average reward or cumulative reward over time. As a result, it is possible to use just enough size for short-term memory in order to reduce the computational time and power necessary where they are crucial factors.

**Figure 8:**
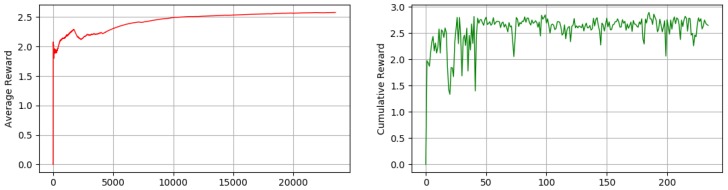
The (**left**) graph shows the average reward that the robot obtains using Deep Actor-Critic (continuous action) over time (showing that robot is able to maximize its average reward). The (**right**) graph shows the cumulative reward that robot accumulate over time and in each episode, the graphs are drawn over (20,000 steps) using no short-term memory.

In continuous-action space, however, we trained our robot over 20,000 steps and calculated the average reward over each 100 steps. As we can see in Figure 8 our robot is able to maximize its average reward over time (Figure 8 (left)) and also maximize and stabilize its cumulative reward over each 100 steps (Figure 8 (right)). One important point regarding the advantage of continuous-action space is that the robot is much more capable in terms of rotation and speed of movement. The capability of rotating in different degrees in different situations makes our robot move to the desire state faster and also without a short-term memory.

**Figure 9:**
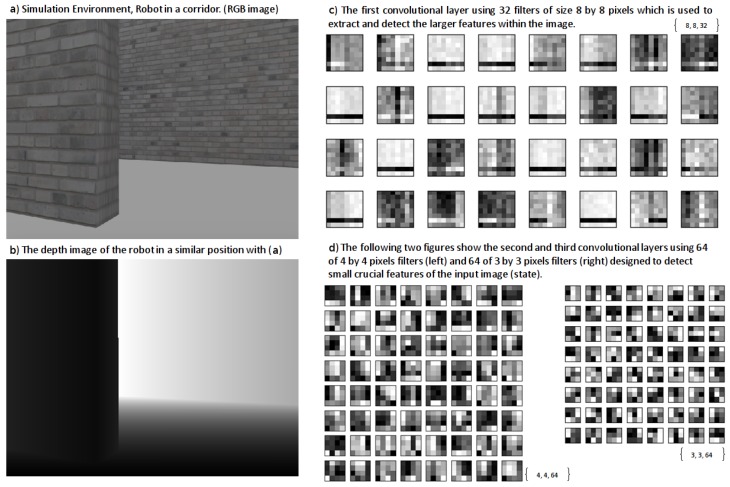
Visualization of our DNN (convolutional neural network).

While the robot is learning, in each step of its movement in the environment, it will receive a perception of its state and tries to take the best action based on its learned model so far. The result of the action it takes will be a reward that will update the model of the robot. Gradually, the robot’s model gets more accurate and its policy will converge to optimal and the robot will be able to autonomously choose the best action to take in different states according to its experiences. A learned state of robot DNN can be seen visualized in Figure 9.

**Figure 10:**
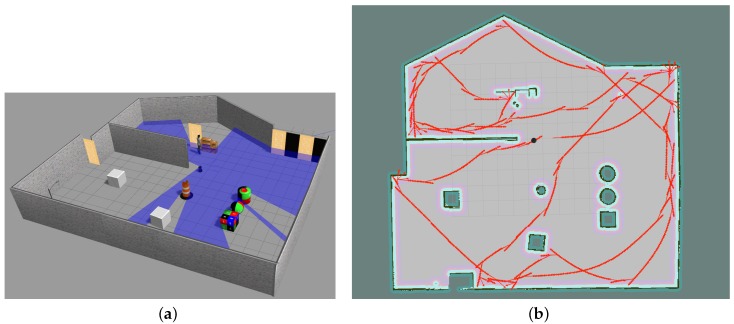
Simulation in a new unknown environment. (**a**) A complex unknown environment simulated in Gazebo; (**b**) SLAM visualization of robot movement in a new unknown complex environment.

As mentioned earlier, we trained our robot in a simple unknown environment first, Figure 6 (left). Figure 6 (center) shows the robot movement using discrete actions after 50,000 steps where it learned how to explore and avoid obstacles efficiently and Figure 6 (right) shows the robot movement using continuous actions after 20,000 steps where it learned how to explore and avoid obstacles efficiently. In order to show our robot movements path we have used SLAM Gmapping ([22]) package (Figure 6 (center) and Figure 6 (right)). Furthermore, results shown in Figure 7 are generated based on the robot movements in the unknown simulated environment shown in Figure 10a and results shown in Figure 8 are generated based on the robot movements in the simple simulated environment shown in Figure 6 (right). After training our robot in the simple unknown environment (Figure 10a, we tested our robot capabilities in a new complex unknown environment (Figure 10b). This new environment is larger compared to the first environment and is more complex considering the different items situated in the environment. The result of robot interaction with this new complex environment can be seen in Figure 10b where it shows that our robot is able to explore and avoid successfully a new unknown complex environment. As our results show, and as we explained in Section 2.3, short-term memory has an important effect on the learning process of the robot while using a discrete-action space; however, it increases the dimensionality of the state and as a result it increases the processing time of the algorithm and its learning time as well.

## 4. Real Test

### 4.1. Environment

As mentioned earlier in the Simulation section, we used ROS deliberately, thus for real tests we have used same nodes, receivers and publishers’ topics that we defined for our simulation. Our ROS is installed on Ubuntu 14. There are different modules defined for our robot, which are depth sensor, range sensors and Kobuki Yujin. These modules are connected to the main algorithm using ROS connectivity protocols. Meanwhile, in the case of our simulation, all modules are defined in Gazebo simulation environment; in our real test scenario, each module is an actual device which is connected to the main algorithm using ROS.

Considering real-world scenarios, sensors encounter a real-world problem, which is noise. In our case, we tackled this problem by design and implementation of our centralized sensor fusion technique alongside a Kalman filter that mitigated the effect of noise in on our system. As a result, our algorithm has a strong and trustable perception in each state.

### 4.2. Robot

**Figure 11:**
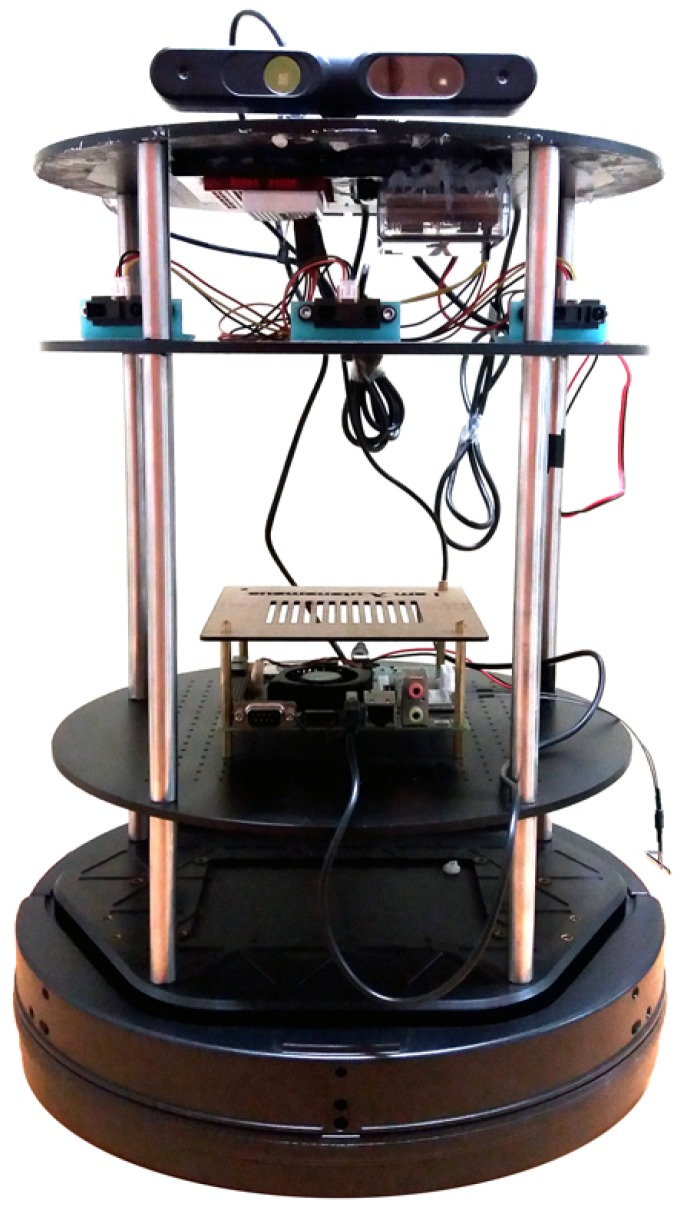
Our simulated robot in Gazebo simulation environment.

Our robot consists of three different sections; sensors, actuators and the brain. The brain of our robot is a Tekra K1 (TK1, Nvidia, Holmdel, NJ, USA), which has Ubuntu and ROS installed, and is connected to its actuators and sensors using USB cables (Figure 11).

#### 4.2.1. Sensors

**Figure 12:**
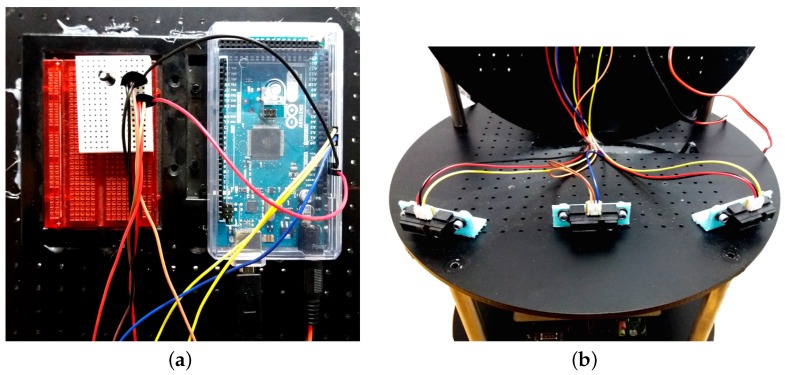
Sensor fusion and state generation. (**a**) An Arduino mega board is used to connect the infra red proximity sensors to Nvidia TK1 board; (**b**) Infra red proximity sensors used as range sensors.

**Figure 13:**
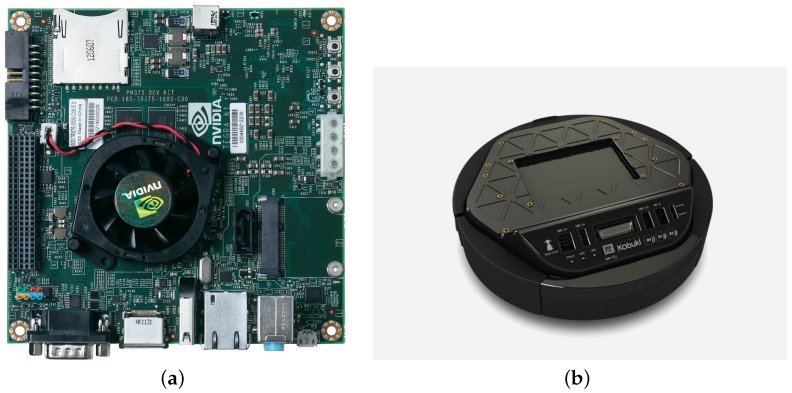
TK1 and Kobuki Yujin. (**a**) Nvidia TK1 board, the Central Processing Part of the Robot; (**b**) Kobuki Yujin, it is the moving platform of the robot and also the energy source for all the modules.

Our robot sensors consist of three range sensors combined with a depth sensor as explained in the Sensor Fusion section. We used the Infrared Proximity Sensor (Sharp GP2Y0A21YK, Osaka, Japan) as our range sensors (Figure 12b) mainly because of its reasonable price compared to other sensors such as LiDAR and its advantage over ultrasonic sensors considering ultrasonic sensors’ problem of reflection. Furthermore, for our range sensors we needed to implement our hardware using an Arduino board (Figure 12a) in order to be able to receive range sensor data in our TK1 board (Figure 13a).

#### 4.2.2. Actuators

The actuator in our robot is its Kobuki Yujin, Seoul, Republic of Korea (Figure 13b), where it is the robot wheels that can move the robot to right, left and forward. Furthermore, Kobuki Yujin is the main energy source for the robot sensors, actuators and brain.

### 4.3. Training and Testing

Considering the robot in a real-world scenario, there are two methods that we can use in order to make our robot able to explore autonomously:

- Train the robot using uninitialized DNN weights: In this scenario, the robot starts training from scratch similar to the simulation case. Implementing this method comes with some difficulties such as the limited energy source of the robot (Robot Battery). Nevertheless, it is crucial for a real robot to have power in order to run all the modules on board. In particular, in our robot we have different modules that are needed to be powered so the robot can execute our algorithm successfully and explore and avoid obstacles autonomously. For example, in our robot we have a TK1 processing unit, 3 Infrared Proximity Sensors, 1 Arduino, Kobuki Yujin, and a depth sensor. All in all, for training our robot using uninitialized DNN weights it needs some time to move around and fill the long-term memory, and using SGD in order to reduce the loss of our q-function and converge to an optimal policy. To solve the issue of power supply there are different possibilities:
  - Software-based solution: It is possible to measure the battery level using internal sensors of Kobuki Yujin, thus, one possibility is to write a procedure for the robot to alarm when the battery is low and stop the training procedure, so a human carer can move the robot and connect it to the power supply, as soon as the power supply level moves above a specific percentage then the robot can resume the training procedure.
  - Hardware-based solution: Adding to the battery cells is one possibility or adding an external battery, but the issue with this method is that it still is possible that the robot turns off in the middle of the training procedure and an external battery can make the robot much heavier. Thus, a better hardware-based solution can be the use of a flexible electrical wire that is hung from the roof and allows the robot to move around for training.
- Using the initialized DNN weights generated in the simulation: By attaching the depth and range sensors in the same position in the real robot that is defined and used in the simulated robot, the DNN weights that are generated by training the robot in a simulated world can be used in the real robot. The benefit of this method is that it can tackle the problem of energy limitation (Battery) and remove the time necessary for training. That being said, it is important to pay attention to two points. Firstly, even though our robot does not do the training step, it still is learning and updating its policy in case there is a change in the environment and this will let the robot to easily move to another unknown environment and adapt itself. Secondly, using DNN weights generated in simulation from scratch for the real robot that uses the same algorithm is totally different to initializing the DNN weights from the beginning in the simulated robot using supervised learning.

In order to test our robot and generate our result we used the second method. We moved the DNN weights that are generated by our MDQL algorithm in the simulation to our real robot in the TK1 module and used them in our real robot for decision-making and adaptation of our MDQL algorithm to the new real-world environment.

### 4.4. Result

**Figure 14:**
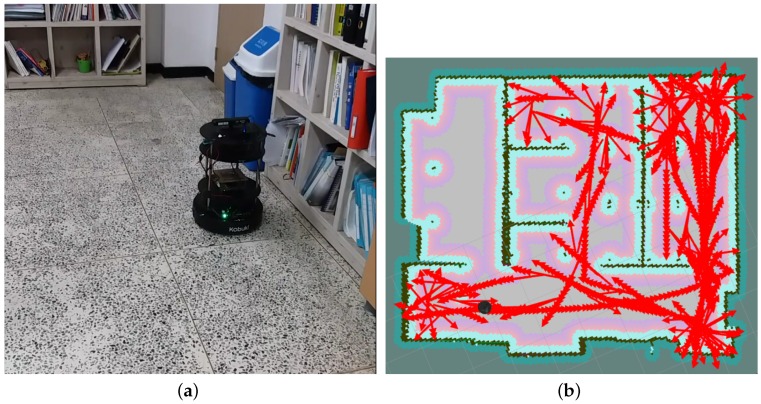
Real test result. (**a**) SLAM visualization of our real robot movement in Smart Autonomous System laboratory; (**b**) Our modified real robot in Smart Autonomous System laboratory.

In order to get the result of our robot exploration in a real environment we used SLAM Gmapping to generates a map of the robot environment using a Hokuyo LiDAR UST-10LX, (Suite A Indian Trail, NC, USA) model sensor connected through ROS protocol by Local Area Network (LAN) cable to our TK1. Furthermore, rviz application was used to draw this information using a mixture of IMU data coming from the Kobuki Yujin in order to draw the odometery information such as moving forward or turning to sides of the robot movement. Nonetheless, it is important to notice that the LiDAR and Inertial Measurement Unit (IMU) sensors are just used for drawing the SLAM Gmapping and not for the purpose of robot movement or decision-making. In other words, our algorithm does not use LiDAR and IMU; their information is just used in on-board processing unit (TK1) for drawing a SLAM map for the purpose of clarifying our robot movement. All the nodes and modules are connected to each other using ROS connectivity protocol and as a result our algorithm implementation is platform-free. The result of our SLAM Gmapping and the odometery information generated in a real environment can be seen in Figure 14b in which our algorithm is driving the robot. We tested our robot in our laboratory (Figure 14a). The exploration path in Figure 14b shows that our robot is able to move between partitions and tables autonomously by avoiding collision with objects.

## 5. Discussion

In this work, we successfully developed a MDLR algorithm capable of choosing discrete or continuous actions—using continuous action space in order to increase the robot’s capabilities in term of its movements and learning—by enriching our previous algorithm [15] and we implemented our work in a simulated and also in a real-world robot. Our results show that our new algorithm is able to learn autonomously and in a continuous way, on-line and from its own experiences to explore its environments in a robust way, and as we demonstrated it can adapt itself to a new environment different from its original environment. In order to robustly recognize the states of our robot and also to tackle the problem of noise in the real world, we used a centralized sensor fusion method which combines our depth sensor with our range sensors. In addition, we tackled the problem of power supply successfully by being able to use the trained weights of our MDRL function approximator generated in the simulation for our real robot. In future works, it should be possible to use information theory-based intrinsic motivations within our algorithm to decrease the search state of the robot and to reach to a new level of intelligence.

## Abbreviations

The following abbreviations are used in this manuscript:

RL	Reinforcement Learning
DRL	Deep Reinforcement Learning
DQN	Deep Q-Network
DNN	Deep Neural Network
ROS	Robot Operating System
SLAM	Simultaneous Localization and Mapping
MDRL	Memory-based Deep Reinforcement Learning
DDPG	Deep Deterministic Policy Gradient
MLP	Multi-Layer Perceptron
